# Genome-Wide Analysis of Caffeoyl-CoA-O-methyltransferase (CCoAOMT) Family Genes and the Roles of *GhCCoAOMT7* in Lignin Synthesis in Cotton

**DOI:** 10.3390/plants13212969

**Published:** 2024-10-24

**Authors:** Lina Ma, Jin Wang, Kaikai Qiao, Yuewei Quan, Shuli Fan, Liqiang Wu

**Affiliations:** 1 Hebei Base of State Key Laboratory of Cotton Bio-Breeding and Integrated Utilization, Hebei Agricultural University, Baoding 071000, China; malina0111@163.com (L.M.); 18232706758@163.com (J.W.); 2 State Key Laboratory of Cotton Bio-Breeding and Integrated Utilization, Institute of Cotton Research, Chinese Academy of Agricultural Sciences, Anyang 455000, China; qiaokaikai@caas.cn; 3 Handan Academy of Agricultural Sciences, Handan 056000, China; quanyuewei@163.com

**Keywords:** cotton, Caffeoyl-CoA-O-methyltransferase (CCoAOMT), gene family, virus-induced gene silencing (VIGS), lignin synthesis

## Abstract

Caffeoyl coenzyme A-O-methyltransferase (CCoAOMT) has a critical function in the lignin biosynthesis pathway. However, its functions in cotton are not clear. In this research, we observed 50 *CCoAOMT* genes from four cotton species, including two diploids (*Gossypium arboretum*, 9, and *Gossypium raimondii, 8*) and two tetraploids (*Gossypium hirsutum*, 16, and *Gossypium barbadense, 17*), performed bioinformatic analysis, and focused on the involvement and functions of *GhCCoAOMT7* in lignin synthesis of *Gossypium hirsutum*. CCoAOMT proteins were divided into four subgroups based on the phylogenetic tree analysis. Motif analysis revealed that all CCoAOMT proteins possess conserved Methyltransf_3 domains, and conserved structural features were identified based on the genes’ exon-intron organization. A synteny analysis suggested that segmental duplications were the primary cause in the expansion of the *CCoAOMT* genes family. Transcriptomic data analysis of *GhCCoAOMTs* revealed that *GhCCoAOMT2*, *GhCCoAOMT7,* and *GhCCoAOMT14* were highly expressed in stems. Subcellular localization experiments of *GhCCoAOMT2*, *GhCCoAOMT7*, and *GhCCoAOMT14* showed that *GhCCoAOMT2*, *GhCCoAOMT7*, and *GhCCoAOMT14* were localized in the nucleus and plasma membrane. However, there are no *cis*-regulatory elements related to lignin synthesis in the *GhCCoAOMT7* gene promoter. *GhCCoAOMT7* expression was inhibited by virus-induced gene silencing technology to obtain gene silencing lines, the suppression of *GhCCoAOMT7* expression resulted in a 56% reduction in the lignin content in cotton stems, and the phloroglucinol staining area corresponding to the xylem was significantly decreased, indicating that *GhCCoAOMT7* positively regulates lignin synthesis. Our results provided fundamental information regarding *CCoAOMTs* and highlighted their potential functions in cotton lignin biosynthesis and lignification.

## 1. Introduction

Lignin, a significant polymeric organic compound in plants, is the second most abundant organic compound after cellulose and a primary constituent of plant cell walls. Its multifaceted contributions to plant growth and development encompass the maintenance of cell wall integrity [1], augmentation of mechanical strength [2], stress resistance [3], heightened pathogen resistance [4], and facilitation of water transport [5,6]. Lignin’s economic significance in agriculture, industry, and commercial applications is substantial. In agriculture, it aids in soil improvement, pesticide substitution, and plant growth promotion, enhancing crop yield and quality. In industry, lignin’s applications are extensive, playing a crucial role in paper manufacturing, fuel and pigment production, and biofuel sectors. In commercial applications, due to its antioxidant and antibacterial properties, lignin is also utilized in the pharmaceutical and food industries. In summary, lignin is not only a vital component of ecological cycles but also provides sustainable raw materials for multiple industries. The main challenges to lignin production in cotton crops are environmental conditions (soil type, temperature, water availability, and stress conditions), the agronomic practices employed in cotton cultivation (fertilizer application and irrigation methods), and cotton varieties exhibit significant genetic variability affecting lignin biosynthesis pathways. The biosynthesis of lignin is a complex biochemical process that involves multiple genes and enzymes.

Lignin is a complex polymer of phenylpropanoid monomers synthesized by the polymerization of three hydroxyl cinnamyl alcohols: p-coumaryl, coniferyl, and sinapyl alcohols. Since lignin has high thermal stability and good adhesion, it can be used as a natural binder for soil improvement. It can be categorized into three types based on the different monomers of lignin: syringyl lignin (S lignin), guaiacyl lignin (G lignin) and hydroxyphenyl lignin (H lignin) [7,8]. O-methyltransferases (OMTs) play key roles in the synthesis of these lignin monomers and are categorized into caffeoyl-coenzyme A O-methyltransferases (CCoAOMTs) and caffeic acid O-methyltransferases (COMTs). While *COMTs* are involved in the syringyl pathway, *CCoAOMTs* participate in the syringyl and guaiacyl pathways [9]. *COMTs*, in coordination with ferulate-5-hydroxylase, catalyze the methylation of 5’-OH on the aromatic ring of lignin aldehydes and lignin alcohols.

*CCoAOMTs* were a class of S-adenosine-L-methionine methyltransferases. They catalyze the transfer of the methyl group of S-adenosine methionine to the benzene ring C3 position of the lignin monomer, converting caffeoyl coenzyme A to feruloyl coenzyme A, providing substrates for syringyl lignin synthesis [10,11,12]. *CCoAOMT* genes encode for CCoAOMT enzymes [13,14]. To date, RNAi, gene knockout, and biological stress have been successfully implemented to modulate lignin content by targeting *CCoAOMT* genes in various plants, affecting plant development and abiotic stress resistance. For example, RNAi was implemented to downregulate the *SmCCoAOMT* of *Salvia miltiorrhiza*, resulting in a significant decrease in lignin content, and affecting the accumulation of phenolic acids [15]. Li et al. demonstrated that lignin concentration in transgenic *maize* could be reduced by 22.4% through downregulation of the *ZmCoA* gene, similarly using RNAi. Histological staining of lignin with Wiesner reagent revealed a slightly higher staining intensity in the xylem and sclerenchyma of RNAi plants than in WT. These results have laid the foundation for the breeding of *maize* with reduced lignin content [16]. Moreover, a *CCoAOMT* gene (*PrCCoAOMT*), involved in the biosynthesis of G lignin in coniferous gymnosperms such as *Pinus radiata*, was successfully cloned. Suppression of this gene in lignin polymers consisting of p-hydroxyphenyl (H), catechyl (C), and guaiacyl (G) subunits [17]. It has been demonstrated that inhibition of *AtCCoAOMT* enhanced the vulnerability of *Arabidopsis thaliana (A. thaliana*) roots to fungal pathogens [18]. Liu et al. investigated the *CCoAOMTs* at the transcriptome level under abiotic stress in *Panicum virgatum*, and showed that *PvCCoAOMT* is involved in lignin biosynthesis and could be highly induced by drought and cold stresses [19]. Wei et al. found that the ABA/MeJA signaling pathway was activated in response to both biotic and abiotic stress via *DfCCoAOMT* genes. They demonstrated that *DfCCoAOMT14* overexpression in the stem of *Dendrocalamus farinosus* resulted in a notable increase in lignin content, xylem thickness, and resistance to drought conditions [20]. *CCoAOMTs* were shown to be linked to the abiotic stress response in jute and are involved in lignin production. Such genes could potentially be utilized to genetically enhance the fiber quality of jute [21]. Liao et al. demonstrated that *CsCCoAOMT1* was involved in the biosynthesis of citrus polymethoxylated flavones [22]. Moreover, it has been reported that *CCoAOMT1* directly acts on the K259 residue that is conserved in *bHLH010* and *bHLH089*, promoting the nuclear localization and function of bHLH transcription factors. This effectively inhibits the expression of downstream genes by reducing the accumulation of bHLHs transcription factors in the nucleus to facilitate pollen development [23]. Following the pioneering discoveries of *CCoAOMT* genes in cell suspension cultures of parsley and carrot by Kuhnl [24] and Pakusch [25], subsequent investigations into the *CCoAOMT* gene family have been conducted in model organisms, such as *A. thaliana* [26], rice [27], *tobacco* [28,29], wheat [30], sorghum [31], and poplar [32] with the increase in the availability of plant genomic data. Studies have demonstrated that the *CCoAOMT* gene family exhibits a high degree of sequence and functional conservation across plant species. For instance, research in tea and *Populus* has revealed the conserved role of this gene family in plant growth, development, and phenylpropanoid metabolism, suggesting that the *CCoAOMT* gene family has undergone stringent selective pressures during plant evolution, maintaining critical biological properties. However, there is limited information available regarding the *CCoAOMT* gene family in cotton, despite its significance as a cash crop.

In this study, the cotton *CCoAOMT* gene family was analyzed using bioinformatics approaches. Their phylogeny, physicochemical properties, gene structure, *cis*-elements in their promoters, chromosome distribution, and collinearity were comprehensively analyzed. Concurrently, the expression profiles of *GhCCoAOMT* gene family members were assessed in different tissues of cotton by employing qRT-PCR analyses, virus-induced gene silencing assays, lignin staining, in conjunction with the quantitative determination of lignin, cellulose, hemicellulose, and pectin. It was found that a reduction in *GhCCoAOMT7* expression in cotton stems resulted in reduced lignin staining in the xylem of cotton stems and a decrease in lignin content, suggesting that *GhCCoAOMT7* was involved in lignin biosynthesis. These findings strongly suggest the involvement of *GhCCoAOMT7* in lignin biosynthesis, thereby enhancing our understanding of *GhCCoAOMT7* functions and laying the foundation for future research on *CCoAOMTs* in cotton.

## 2. Results

### 2.1. Identification and Phylogenetic Analysis of the CCoAOMT Family Members in Cotton

In total, 16 *CCoAOMT* genes were identified in *Gossypium hirsutum* (*G. hirsutum*), 17 in *Gossypium barbadense* (*G. barbadense)*, 9 in *Gossypium arboretum* (*G. arboretum*), and 8 in *Gossypium raimondii* (*G. Raimondii*). All these identified *CCoAOMT* genes were named by the chromosomal locations. The information for these identified *CCoAOMT* genes was summarized in Table A1, including chromosome location, isoelectric point (pI), amino acid length, molecular weight (MW), and projected subcellular localization. The pI, MW, and protein length of the projected CCoAOMT proteins varied from 11.91 to 36.2 kDa, 4.42 to 9.69, and 108 to 318 amino acids, respectively.

**Figure 1:**
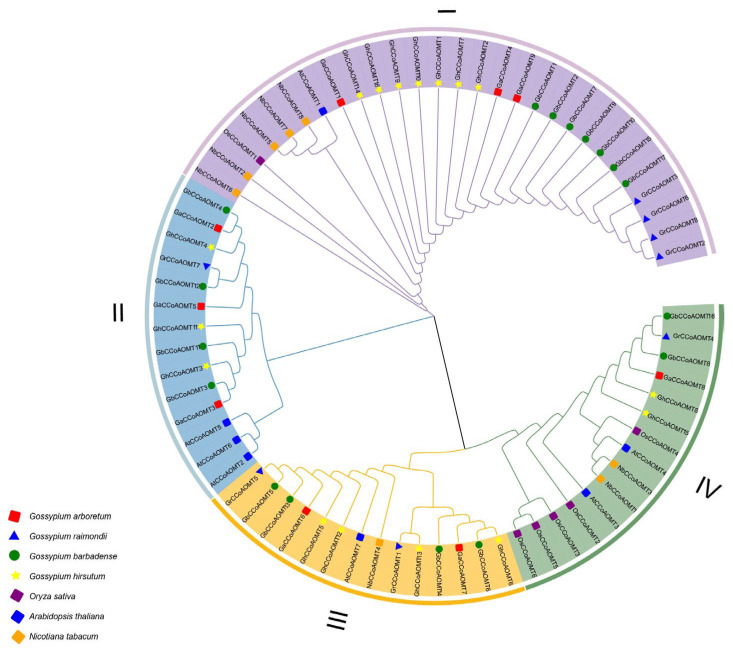
Phylogenetic relationship of CCoAOMTs proteins from *Gossypium hirsutum*, *Gossypium barbadense*, *Gossypium arboretum*, *Gossypium raimondii*, *Oryza sativa*, *Nicotiana tabacum*, and *Arabidopsis thaliana*.

The phylogenetic tree, which revealed the evolutionary relationships between CCoAOMT proteins from multiple species, included a total of 71 full-length proteins from *G. raimondii* (8), *G. arboretum* (9), *G. barbadense* (17), *G. hirsutum* (16), *Oryza sativa* (6), *N. tabacum* (8), and *A. thaliana* (7) (Figure 1). The seventy-one CCoAOMT proteins from these seven species were clustered into four branches. Twenty-eight CCoAOMTs were clustered in group I (four GrCCoAOMTs, seven GbCCoAOMTs, three GaCCoAOMTs, seven GhCCoAOMTs, five NbCCoAOMTs, AtCCoAOMT1, and OsCCoAOMT1). Fourteen CCoAOMTs were clustered in group II, including GrCCoAOMT7, three AtCCoAOMTs, three GaCCoAOMTs, three GhCCoAOMTs, and four GbCCoAOMTs. Additionally, fourteen CCoAOMTs were clustered in group III, including two GaCCoAOMTs, two GrCCoAOMTs, four GbCCoAOMTs, four GhCCoAOMTs, AtCCoAOMT7, and NbCCoAOMT14. Fifteen in group IV, including five OsCCoAOMTs, two GbCCoAOMTs, two GhCCoAOMTs, two NtCCoAOMTs, two AtCCoAOMTs, GaCCoAOMT8, and GrCCoAOMT4.

### 2.2. CCoAOMTs Family Motif and Gene Structure Analysis

**Figure 2:**
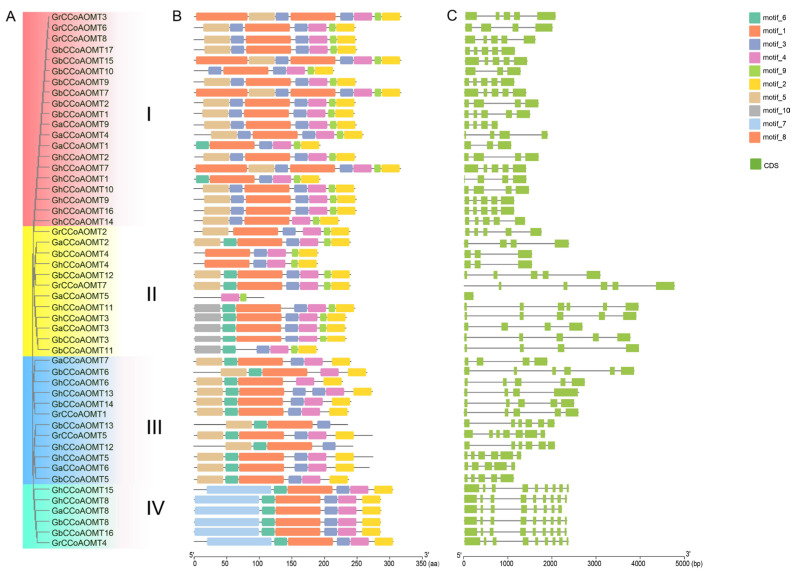
Comprehensive analysis of the structural properties of the cotton *CCoAOMT* genes family. (**A**) Phylogenetic tree of the cotton *CCoAOMT* genes family. (**B**) Conserved motif of the four cotton CCoAOMT proteins. (**C**) Gene structure of the four cotton CCoAOMT proteins.

The *CCoAOMT* gene family member properties were more thoroughly assessed through a combined phylogenetic tree, gene structure, and motif analysis. According to the clustering of the *CCoAOMT* gene family in the five examined species, the *CCoAOMT* gene family members in the four cotton species were divided into four groups: I, II, III, and IV (Figure 2A). As is shown in Figure 2B, all *CCoAOMTs* in group I contained two copies of motifs 3. The *CCoAOMTs* in Group II contained a single motif 6, and some *CCoAOMTs* included motif 10, which was absent in other genes from other groups, such as *GhCCoAOMT3*, *GaCCoAOMT3*, *GbCCoAOMT3*, *GhCCoAOMT11*, and *GbCCoAOMT11*. Group III included relatively similar but diverse motif structures, such as motif 5, motif 4, motif 1, motif 8, motif 3, motif 7, and motif 2, arranged in the order of most frequently present to less frequently present. Group IV members possess motif 7, which was absent in other classes. Overall, motifs 1–4 were postulated to play crucial roles in keeping the structural stability and gene functional specificity of CCoAOMT proteins. Furthermore, using the Gene Structure Display Server 2.0 to plot the gene structures (Figure 2C). The results clearly revealed that the exon numbers significantly varied between the *CCoAOMT* gene family members from one to nine, which may be related to splicing site mutations. We also found that all paralogs present in the same branch of the phylogenetic tree shared similar numbers of exons/introns.

### 2.3. Chromosomal Distribution and Synteny Analysis of CCoAOMTs

**Figure A1:**
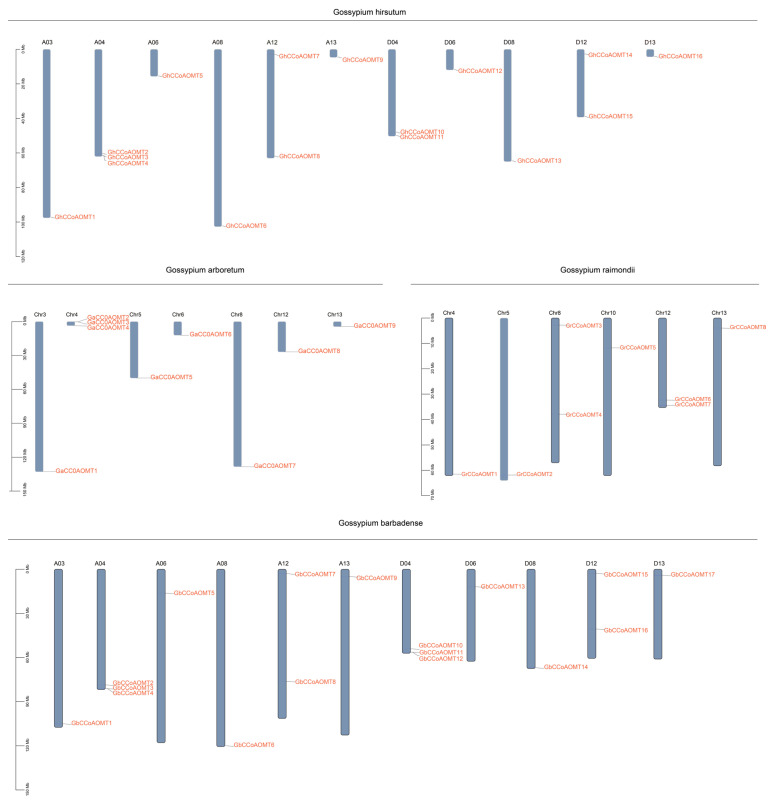
Chromosomal distribution of *CCoAOMTs* genes in cotton.

The chromosomal locations of the *CCoAOMTs* were determined in the chromosomes of the At and Dt cotton sub-genomes (Figure A1). In *G. arboretum*, the nine *GaCCoAOMTs* were mainly distributed on seven chromosomes, namely Chr3, Chr4, Chr5, Chr6, Chr8, Chr12, and Chr13. Only Chr4 contained three *GaCCoAOMTs*, and the rest of the chromosomes contained only one *GaCCoAOMT*. In *G. raimondii*, six *GrCCoAOMTs* were distributed on six chromosomes, namely Chr4, Chr5, Chr8, Chr10, Chr12, and Chr13. In *G. hirsutum*, a total of 16 *GhCCoAOMTs* were located in the 11 chromosomes. While three *GhCCoAOMTs* were located in the A04 chromosome. In *G. barbadense*, a total of 17 *GbCCoAOMTs* were distributed on 11 chromosomes, with three *GbCCoAOMTs* located on the A04 and D04 chromosomes. On the contrary, no *CCoAOMT* genes are present on the rest of the chromosomes, such as Chr1, Chr2, Chr7, Chr9, and Chr11. The uneven chromosomal distribution of *CCoAOMT* genes indicated significant genetic diversification during the evolutionary process.

**Figure 3:**
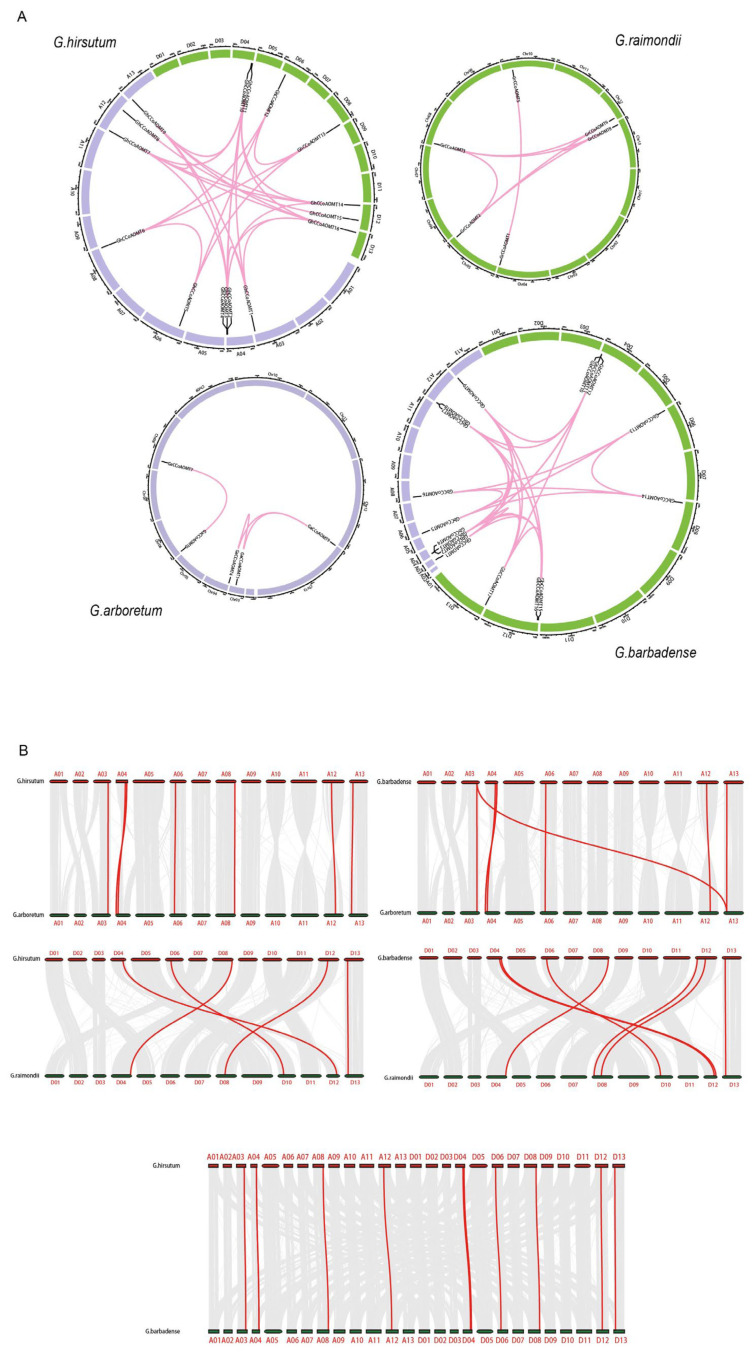
Synteny analysis of the *CCoAOMT* genes. (**A**) Genomic synteny analysis of *GaCCoAOMTs*, *GrCCoAOMTs*, *GhCCoAOMTs*, and *GbCCoAOMTs*. Purple and green blocks represent the A-subgenome and D-subgenome, respectively. Numbers along each chromosome block represent the sequence lengths in megabases. Red lines indicate the duplicated *CCoAOMT* pairs. (**B**) Synteny and collinearity relationships between *G. hirsutum* and *G. arboreum*, between *G. hirsutum* and *G. raimondii*, between *G. barbadense* and *G. arboretum*, between *G. barbadense* and *G. raimondii*, and between *G. hirsutum* and *G. barbadense*. Gray lines depict the collinearity between different genomes, and red lines depict the collinearity of the *CCoAOMT* genes between different genomes.

Three processes are often involved in the evolution of gene families: tandem duplication, whole genome duplication, and fragment segmental duplication [33]. Detection of gene duplication events in the genomes of upland cotton using MCScan X revealed that all 4 *GaCCoAOMT* gene pairs in *G. arboretum*, all 7 pairs of *GrCCoAOMT* genes in *G. raimondii*, all 19 pairs of *GbCCoAOMT* genes in *G. barbadense*, and all 26 pairs of *GhCCoAOMT* genes in upland cotton were derived from segmental duplications. This indicated that segmental duplications were the primary mode of the *GhCCoAOMT* gene family expansion in upland cotton (Figure 3A). In addition, a total of 39 duplicated gene pairs were identified among the 50 cotton *CCoAOMT* genes. Computed the Ka/Ks ratios between *CCoAOMT* duplicate gene pairs. The results showed that only the KA/KS ratios of *GhCCoAOMT6*—*GaCCoAOMT7* were greater than one. Meanwhile, the Ka/Ks ratios of the other 38 *CCoAOMT* gene pairs investigated were below one, suggesting that purifying selection was applied to them (Table A2).

Allotetraploid species, *G. hirsutum* and *G. barbadense*, were produced by the hybridization between diploid A and D genome species [34]. Thus, we performed a collinearity analysis of the A and D genomes between the *G. hirsutum* and *G. barbadense* with *G. arboretum* and *G. raimondii* (Figure 3B). A high collinearity was observed at the whole-genome level between *G. barbadense* and *G. hirsutum* with their diploid progenitors. Furthermore, seven of the nine *CCoAOMT* genes in the A genome of upland cotton were highly collinear with those of *G. arboretum*, and five of the seven genes in the D genome were highly collinear with those of *G. raimondii*, indicating that the other two genes (*GhCCoAOMT11* and *GhCCoAOMT16*) were lost during the tetraploidization of *G. hirsutum*. Six of the nine genes in the A genome of *G. barbadense* were highly collinear with those of *G. arboretum*, and seven of the eight genes in the D genome of *G. barbadense* were highly collinear with those of. *G. raimondii*, indicating that the other three genes (*GbCCoAOMT6*, *GbCCoAOMT11*, and *GbCCoAOMT15*) were lost during the tetraploidization of *G. barbadense*. Fiftenn genes of *G. hirsutum* were highly collinear with those of *G. barbadense*, speculating that *GhCCoAOMT14* was formed after the genome duplication.

### 2.4. Cis-Element Analysis of CCoAOMTs

**Figure 4:**
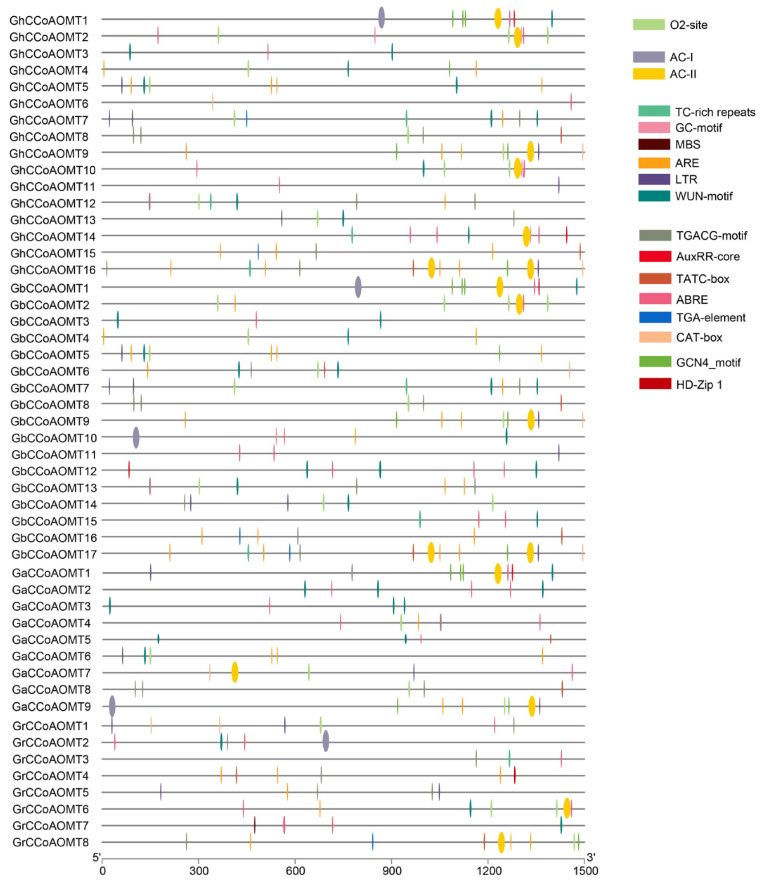
*Cis*-elements present in the *CCoAOMTs* promoters.

*Cis*-elements in the promoter region upstream of a gene often control the expression of that gene. Promoters of putative *CCoAOMT* genes were identified and examined for the presence of *cis*-elements to analyze the potential involvement of *CCoAOMT* genes in the responses to various conditions. In this study, we examined the promoter region 1500 bp upstream of the *CCoAOMT* transcription start sites (ATG codon). The findings revealed that in addition to core elements such as TATA-box, O2-site, and other commonly present elements, the promoter of the *CCoAOMT* genes contained 17 additional *cis*-element types (Figure 4, Table A3). Most were light signal transduction-related elements, such as AE-Box, ACE, GATA-motif, etc. There were plant hormone-responsive elements involved in Methyl Jasmonate (TGACG-motif), auxin (AUXRR-core and TGA-element), gibberellin (TATC-box), and abscisic acid (ABRE) signal transduction and responses. Elements associated with plant growth and development, such as meristem development (CAT-box), endosperm development (GCN4-motif), and palisade cell differentiation (HD-zip). Various stress-responsive *cis*-elements were also discovered, such as defense and wound response (TC-rich), drought response (MBS), low-temperature response (LTR), and plant hypoxia and anaerobic response (GC-motif and ARE) elements. In addition, we found that most *CCoAOMTs* in group I contained the AC-I or AC-II cis-element, except for *GhCCoAOMT7*, *GaCCoAOMT4*, and *GbCCoAOMT15*. AC-I and AC-II are important *cis*-elements involved in plant lignin synthesis [35,36,37], suggesting that the lignin production may be connected to these genes.

### 2.5. Subcellular Localization of CCoAOMT Proteins

**Figure 5:**
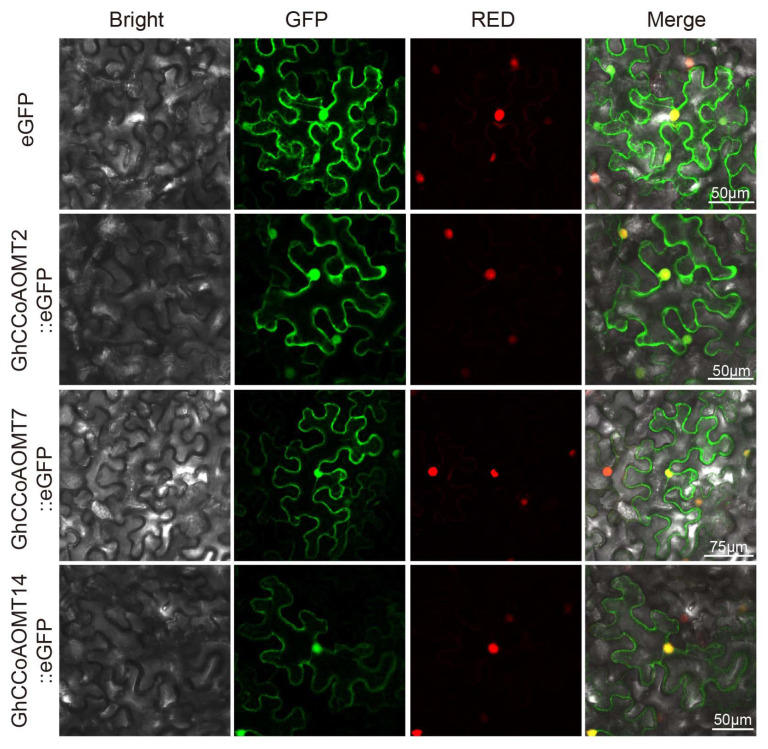
Subcellular localization of CCoAOMT proteins.

To further study the functions of CCoAOMT proteins, the subcellular localization of CCoAOMT family members was predicted using the CELLO v.2.5 online tool. Based on the predicted subcellular localization, twenty-nine of the CCoAOMT proteins were exclusively localized into the cytoplasm, five CCoAOMT proteins were localized in the chloroplast, one CCoAOMT protein was localized in the mitochondrion, four CCoAOMT proteins were localized in the nucleus, and eleven CCoAOMT proteins were predicted to be localized in the cytoplasm, plasma membrane, chloroplasts, and mitochondria (Table A1). Generally, a gene’s function correlates with its corresponding protein’s subcellular location [38,39]. We generated pGhCCoAOMT2::eGFP, pGhCCoAOMT7::eGFP, and pGhCCoAOMT14::eGFP vectors and expressed these fusion proteins in *N. tabacum* leaf cells. The pGhCCoAOMT2::eGFP, pGhCCoAOMT7::eGFP, and pGhCCoAOMT14::eGFP fusion vector’s fluorescence signal was detected in the nucleus and plasma membrane. In contrast, the empty vector eGFP protein displayed a fluorescence signal in the cytoplasm, plasma membrane, and nucleus, indicating that GhCCoAOMT2, GhCCoAOMT7, and GhCCoAOMT14 were located in the nucleus and plasma membrane (Figure 5).

### 2.6. Expression Analysis of the GhCCoAOMT Genes in Different Tissues in G. hirsutum

**Figure 6:**
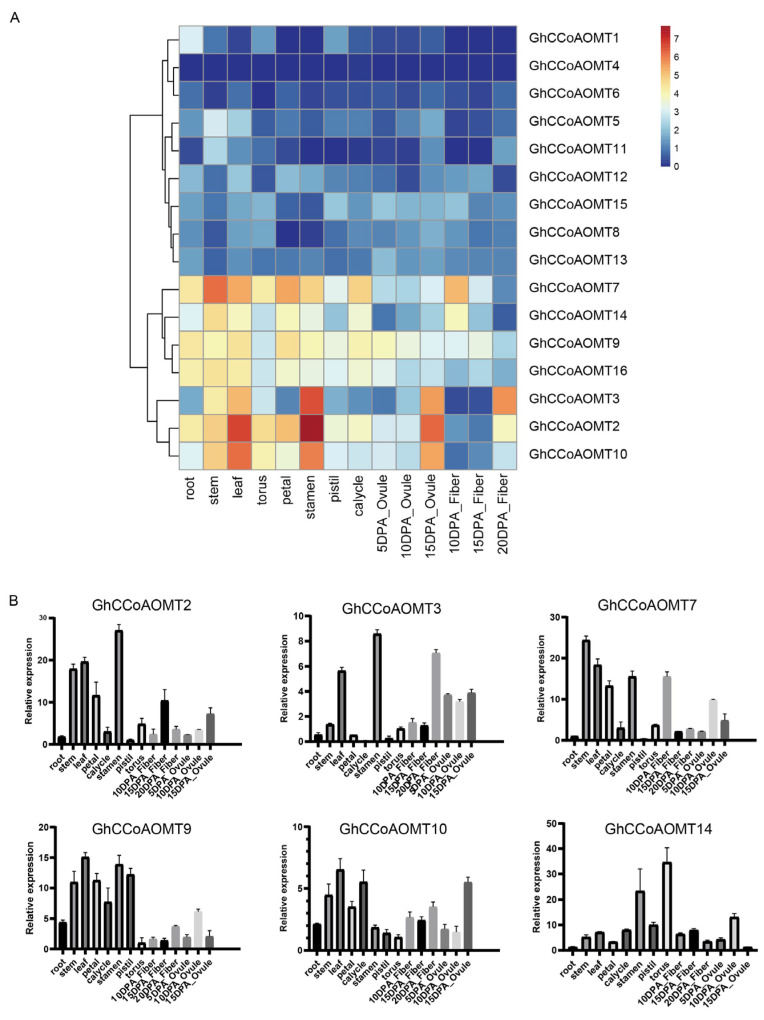
Expression patterns of *CCoAOMTs* in (**A**) ten plant tissues (root, stem, leaf, torus, petal, stamen, pistil, calycle, ovule, fiber). Color scale represents FPKM normalized log2 transformed counts where blue represents low expression and red represents high expression. (**B**). Relative expression of *GhCCoAOMT2*, *GhCCoAOMT3*, *GhCCoAOMT7*, *GhCCoAOMT9*, *GhCCoAOMT10*, and *GhCCoAOMT14* in different tissues assessed by qRT-PCR and calculated by the 2^−ΔΔCt^ method. Mean values and standard deviations were obtained from three technical replicates and biological replicates. Error bars represent the standard deviation estimated by three independent experiments.

The spatiotemporal gene expression patterns greatly correlate with the gene’s biological function. The expression patterns of *GhCCoAOMT* genes were analyzed to explore their roles in cotton growth and development. The expression of most *GhCCoAOMTs* was low in various tissues. *GhCCoAOMT1*, *GhCCoAOMT4,* and *GhCCoAOMT11* were not expressed in almost all tissues. Certain *GhCCoAOMT* genes, such as *GhCCoAOMT2*, *GhCCoAOMT3*, *GhCCoAOMT7*, *GhCCoAOMT9*, *GhCCoAOMT10*, *GhCCoAOMT14*, and *GhCCoAOMT16*, were expressed in all tissues. Among them, *GhCCoAOMT2* was highly expressed in stems, leaves, stamen, and 15dpa ovules, especially in the stamen. *GhCCoAOMT7* was most highly expressed in stems. *GhCCoAOMT10* was highly expressed in leaves, stems, stamens, and 15dpa ovules (Figure 6A). In addition, we selected several genes expressed in various tissues for qRT-PCR analysis to validate the accuracy of these results. The qRT-PCR results were highly consistent with those mentioned above (Figure 6B). These findings provide a reference for our further research on the biological function of *GhCCoAOMTs*.

### 2.7. Silencing of GhCCoAOMT7 Affects Lignin Synthesis in Cotton

According to the analysis of family gene expression, *GhCCoAOMT7* was highly expressed in stems. Although it belongs to group I, its promoter is the only promoter that does not contain AC-I or AC-II *cis*-elements related to lignin synthesis. Therefore, we selected the *GhCCoAOMT7* gene for further study to determine whether it is related to lignin biosynthesis.

**Figure 7:**
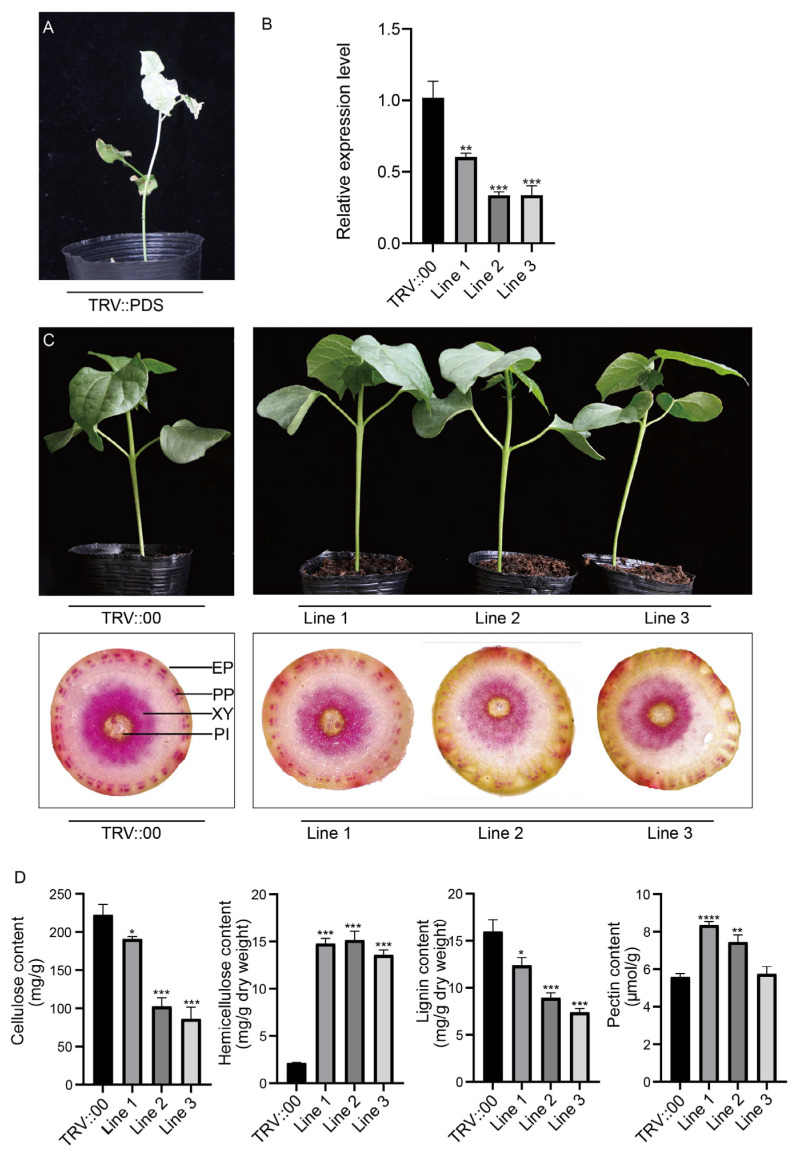
Silencing of *GhCCoAOMT7* reduces cotton stem lignin content. (**A**) corresponds to the TRV::PDS positive control. (**B**) Relative expression of *GhCCoAOMT7* in the cotton stem was assessed by qRT–PCR and calculated by the 2^−ΔΔCt^ method. Mean values and standard deviations were obtained from three biological and three technical replicates (n = 9). Error bars indicate the standard deviation estimated by three independent experiments. (**C**) TRV::00 and TRV::GhCCoAOMT7 silenced cotton plant growth statues and stem phloroglucinol staining. EP, Epidermis; PP, Phloem fibers; XY, Secondary xylem; PI, Pith cells. (**D**) Determination of cellulose, hemicellulose, lignin, and pectin in the TRV::00 and TRV::GhCCoAOMT7 cotton stems. Asterisks (* *p* < 0.05; ** *p* < 0.01; *** *p* < 0.001; and **** *p* < 0.0001) indicate significant differences from the blank control (pTRV:00).

The TRV::GhCCoAOMT7 vector was generated to downregulate the expression of *GhCCoAOMT7*, and it was transformed into Agrobacterium. Three weeks post agroinfiltration, the TRV::PDS vector resulted in an albino phenotype, indicating the success of the VIGS assay (Figure 7A). To further evaluate the VIGS assay’s effectiveness, we utilized qRT-PCR to detect the expression levels of *GhCCoAOMT7* in plants transformed with the TRV::GhCCoAOMT7 and TRV:00 vectors. As demonstrated in Figure 7B, the expression level of *GhCCoAOMT7* was significantly decreased in TRV::GhCCoAOMT7-treated plants, implying that the gene had been successfully silenced. Then, the stems from the TRV::GhCCoAOMT7 and TRV::00 cotton plants were collected for chemical phloroglucinol staining. The lignin staining results indicated that the TRV::GhCCoAOMT7 cotton exhibited a reduced and low-intensity cross-section staining in comparison to the TRV::00 plants (Figure 7C).

The cell wall of cotton is mainly composed of cellulose, hemicellulose, lignin, and pectin. In order to determine these components, the stems of TRV::00 and TRV::GhCCoAOMT7 cotton plants were collected. As shown in Figure 7D, the content of cellulose and lignin in stems decreased significantly in the TRV::GhCCoAOMT7 plants, while the content of hemicellulose increased. However, pectin content did not change significantly, which may be due to the compensatory regulatory function between lignin and cellulose. Those results suggested that the *GhCCoAOMT7* gene may be involved in lignin biosynthesis.

## 3. Discussion

The primary constituent of the cell wall, lignin, plays key roles in the plant’s responses to both biotic and abiotic stressors [12]. The functions of the *CCoAOMT* gene family in the lignin synthesis pathway have been studied in *A. thaliana* [26], sorghum [31], and other plants. In this research, we used bioinformatics tools to systematically identify and characterize the cotton *CCoAOMT* family genes, and focus on validating the role of *GhCCoAOMT7* in cotton.

### 3.1. Characterization of CCoAOMTs in Cotton

Allotetraploid cotton genetic resources were obtained by interspecific hybridization between *G. arboretum* and *G. raimondii* [40]. While *G. barbadense* D-subgenomes and *G. hirsutum* D-subgenomes source from the genome of *G. raimondii* [39], the *G. barbadense* A-subgenome, and *G. hirsutum* A-subgenome descended from common descent, *G. arboretum* [37,40]. This study identified 9, 8, 17, and 16 *CCoAOMT* genes in *G. arboreum*, *G. raimondii*, *G. barbadense*, and *G. hirsutum*, respectively. These were clustered based on the phylogenetic analysis into four groups. Analysis of *CCoAOMT* gene duplications indicated that segmental duplications are the primary mode of expansion in cotton for the *CCoAOMT* gene family. Four cotton species were used to further study the Ka and Ks and the Ka/Ks ratios of *CCoAOMT* segmentally duplicated gene pairs. The Ka/Ks ratios of most of the duplicated gene pairs of *CCoAOMT* in cotton were less than one, and *CCoAOMT* genes in four cotton species had undergone purifying selection during the evolutionary process [41]. Among all sixteen *GhCCoAOMTs* of *G. hirsutum*, nine *GhCCoAOMTs* were located in the *G. hirsutum* A-subgenom, and seven *GhCCoAOMTs* in the *G. hirsutum* D-subgenome. Similarly, in *G. barbadense*, nine *GbCCoAOMTs* were located in the *G. barbadense* A-subgenome, and eight *GbCCoAOMTs* were located in the *G. barbadense* D-subgenome. The above observations indicated that *CCoAOMTs* of the D genome appear to be lost to varying degrees during cotton polyploidization. This finding not only deepens our understanding of the genomic structure and evolution of cotton, but also provides a basis for further research into the functions and regulatory mechanisms of the *CCoAOMT* genes. Further analysis of the distribution and evolutionary features of these genes revealed significant differences in the number of *CCoAOMT* genes in the different subgenomes, indicating dynamic changes in the different subgenomes during evolution.

### 3.2. Regulatory Mechanisms and Functions of CCoAOMTs

The function of related gene families can be studied more precisely by gene structure analysis, phylogenetic analysis, and their subcellular localization [42]. The cotton *CCoAOMTs* gene structure differed greatly, consisting of a maximum of nine exons and a minimum of just one exon. The varied exon-intron architectures can lead to gene function diversification [43]. The gene structure analysis of the *CCoAOMT* family in cotton was carried out using the acquired NWK data and motif files. Although the motif types in each class were almost identical, individual genes were structurally unique from other *CCoAOMT* genes. The comparatively conserved function of the *CCoAOMT* genes potentially derived from the presence of the highly conserved motifs 1–4 found in cotton. Additional motifs can be gradually incorporated or removed throughout evolution to alter the functions of genes in plants. By subcellular localization analysis of cotton CCoAOMT proteins, it was found that most proteins were located in the cytoplasm, certain proteins were predicted to be located in the cytoplasm, plasma membrane, chloroplasts, and mitochondria, and a few proteins were located in the nucleus. We performed an in vivo subcellular localization study of *GhCCoAOMT2*, *GhCCoAOMT7*, and *GhCCoAOMT14* through their transient expression in *N. benthamiana* leaf cells. We determined that GhCCoAOMT2, GhCCoAOMT7, and GhCCoAOMT14 were localized in the nucleus and the plasma membrane. This is the first time *CCoAOMTs* have been observed to be localized to plants’ nucleus and plasma membrane since their discovery, possibly due to differences between plant species.

The promoter sequence upstream of the transcription start site houses *cis*-elements that control downstream gene expression in response to various biotic or abiotic stressors in order to adjust to various growth conditions [44]. Plant hormone responsive *cis*-elements and other *cis*-elements linked to stress response were found in the promoters of *CCoAOMT* gene family members. Every gene contained a different set of *cis*-elements that were involved in plant growth and development regulation or resistance to stress. In this study, we analyzed the expression of identified *GhCCoAOMT* family genes in different cotton tissues using transcriptome data and validated the results with qRT-PCR experiments. We found that *GhCCoAOMT14* in the torus showed opposite expression patterns in the transcriptome and qRT-PCR results. This discrepancy is probably due to technical differences. RNA-Seq captures the expression patterns of the entire transcriptome, including low and medium abundance transcripts, while qRT-PCR is a quantitative technique that targets specific loci and genes and requires highly purified RNA samples with strict quality control during RNA extraction and reverse transcription. RNA-Seq is more sensitive for detecting low-abundance transcripts and may identify transcripts that are too low in abundance to be fully detected by qRT-PCR [45,46]. Therefore, we consider the transcriptome results as the primary reference in this study.

### 3.3. The Function of GhCCoAOMT7 in Cotton

Our study provides insights into the role of *GhCCoAOMT7* in lignin biosynthesis in cotton stems and emphasizes the importance of this gene as a potential breeding target. The results suggest a close correlation between the high expression of *GhCCoAOMT7* in stems and lignin biosynthesis, consistent with its clustering in the phylogenetic tree. Although the promoter of *GhCCoAOMT7* does not contain typical *cis*-elements associated with lignin biosynthesis (such as AC-I or AC-II) [35,36], its crucial role in lignin synthesis was experimentally confirmed [37]. Through gene silencing experiments, we observed a significant decrease in lignin biosynthesis in the transgenic cotton plants (TRV::GhCCoAOMT7), characterized by a reduction in the staining area and intensity of the xylem. These phenomena suggest that *GhCCoAOMT7* plays a key role in the lignin biosynthesis process in cotton. In addition, quantitative analysis of cell wall components indicated changes in the content of cellulose, hemicellulose, and lignin, further supporting the functional contribution of *GhCCoAOMT7*. This finding not only reveals the involvement of *GhCCoAOMT7* in lignin synthesis but also provides important insights into the complex mechanisms coordinating the synthesis of lignin components. The investigation of *GhCCoAOMT7* offers new molecular targets for genetic improvement and breeding strategies in cotton. Increasing lignin content can increase plant stress resistance and improve fiber quality in cotton production [38]. Therefore, precise regulation of *GhCCoAOMT7* expression could facilitate the optimization of lignin biosynthesis and thus improve cotton growth and yield. Future research should further investigate the interactions between *GhCCoAOMT7* and other genes related to lignin biosynthesis, as well as its functional variations under different environmental conditions.

In summary, this study confirms the significant role of *GhCCoAOMT7* in lignin biosynthesis in cotton, laying a foundation for further exploration of the mechanisms involved in lignin synthesis and its applications in cotton breeding. This not only deepens our understanding of the biological functions of *CCoAOMT* genes in plant growth and development but also provides theoretical support for future strategies in cotton improvement.

## 4. Materials and Methods

### 4.1. Identification and Phylogenetic Analysis of CCoAOMT Proteins in Cotton

The genomes of four different cotton species, two diploids (*G. arboretum*, CRI; *G. raimondii*, JGI), and two allotetraploids (*G. hirsutum*, NBI; *G. barbadense*, ZJU) were download from the online database CottonFGD (https://cottonfgd.net/) [47]. The *A. thaliana, Oryza sativa*, and *N. tabacum* CCoAOMT family protein sequences were downloaded from the *A. thaliana* genome database (https://www.arabidopsis.org/), and NCBI (https://www.ncbi.nlm.nih.gov/). The hidden Markov model profile of the Methyltransf_3 domain (PF01596) was obtained to search for all CCoAOMT proteins in the four cotton species genomes (https://www.ncbi.nlm.nih.gov/). The Pfam (https://pfam.xfam.org/) and SMART website(http://smart.embl-heidelberg.de/) were used to confirm that the predicted proteins contained the Methyltransf_3 domain [48]. Alignment of *A. thaliana*, *G. raimondii*, *G. arboretum*, *G. barbadense*, *G. hirsutum, Oryza sativa*, and *N. tabacum* CCoAOMT protein domain sequences was conducted using the MUSCLE method of MEGA7.0 with the default parameters [49]. Using the neighbor-joining (NJ) approach, phylogenetic analysis was performed on the aligned sequences. Using MEGA 7.0 and the P-distance model, a pairwise deletion option, and 1000 bootstrap repetitions, the consensus tree was built [50]. Finally, Adobe Illustrator CS6 (Version 16) software was used to more clearly illustrate the subfamilies and visually edit the phylogenetic trees.

### 4.2. Bioinformatics Analysis of CCoAOMT Proteins in Cotton

The program ExPASy was utilized to calculate the isoelectric point (pI) as well as the molecular weight (MW) of each of the CCoAOMT proteins [51,52]. CELLO v.2.5 was used for subcellular localization predictions [39]. The data of the cotton genome chromosomes were procured from the online repository CottonFGD (https://cottonfgd.net/). The Multiple Collinearity Scan tool was employed to examine synteny and collinearity [53]. Using the TBtools Version 1.115, the non-synonymous (Ka) and synonymous (Ks) substitution rates were computed to estimate the selection pressure acting on the cotton *CCoAOMTs* during their evolution. Positive, neutral, and purifying selection are generally indicated by Ka/Ks > 1, Ka/Ks = 1, and Ka/Ks < 1 [54]. Using the OmicStudio to create the circos plot and label the chromosomal locations of the CCoAOMT proteins [55]. Gene structure data of the *CCoAOMT* gene family members were downloaded from the online website CottonFGD, and the GSDS2.0 web server (https://gsds.gao-lab.org/) was used to graphically examine their gene structures [56]. The web program MEME predicted the conserved motifs of the proteins of all gene family members encoded. The findings were visualized using TBtools, and the maximum number of motifs was set to 10. The remaining parameters were left at their default values. Upstream promoter region sequences (1500 bp) of all *CCoAOMTs*, starting from the initiation codon (ATG), were retrieved. The PlantCARE database was used to identify the cis-elements within these sequences [57].

### 4.3. Expression Pattern Analysis

Based on the *GhCCoAOMT* gene ID in *G. hirsutum* identified by the gene family evolutionary tree, transcriptome data were obtained from different cotton tissues (root, stem, leaf, torus, petal, stamen, pistil, calycle) and developmental stages (5, 10, 15 DPA ovule, and 10, 15, 20 DPA fiber) retrieved from CottonFGD (https://cottonfgd.net/) to gain further insights into the expression patterns of *GhCCoAOMTs*. TBtools Version 1.115 software was used to visualize the heatmap of the *GhCCoAOMTs* expression and FPKM normalized log2 transformed counts.

### 4.4. Subcellular Localization Experiments

Using the primers listed in Table A4, the coding sequences of *GhCCoAOMT2*, *GhCCoAOMT7*, and *GhCCoAOMT14* were extracted from CCRI24 cDNA by PCR, and the resulting fragments were ligated to the pCAMBIA-2300-35S::eGFP vector to form the pCAMBIA-2300-35S::GhCCoAOMT7-eGFP vector. The recombinant vector was transfected into the Agrobacterium tumefaciens strain GV3101, which was then injected into tobacco leaves to be transiently expressed. The inoculated tobacco plants were grown in 12 h of darkness and 24 h of light. An aDmi8 inverted phase microscope (Leica) was used to observe the resulting eGFP fluorescence.

### 4.5. Plant Material and Virus-Induced Gene Silencing

The recombinant vector pTRV::GhCCoAOMT7 was constructed by inserting a 300 bp fragment of *GhCCoAOMT7* into the pTRV2 vector. Separate transformations of the vectors containing the recombinant vector (pTRV::GhCCoAOMT7), the positive control vector (pTRV::PDS), and the empty vector (pTRV::00) were carried out in GV3101 Agrobacterium Competent Cells. Cotton seedlings whose cotyledons have just begun to flatten were selected and were watered prior to the agroinfiltration. A tiny puncture was created in the cotyledon’s epidermis using a sterile needle, and then a syringe was used to inject the bacterial solution into it until the entire cotyledon was filled with the solution. The CCRI24 seedlings were kept in the dark for 24 h following agroinfiltration. All of the seedlings were then moved to a greenhouse where they were cultured under 16 h of light and 8 h of darkness at 25 °C.

### 4.6. RNA Extraction and qRT-PCR Detection

The samples’ total RNA was extracted using the fast pure plant total RNA isolation kit (Vazyme Biotech, Nanjing, China). Transcript all-in-one first-strand cDNA synthesis supermix (TransGen Biotech, Beijing, China) for qPCR was used to create the cDNA from the reverse transcription of RNA. ABI 7500 real-time PCR equipment (Applied Biosystems, Foster City, CA, USA) was used to carry out qRT-PCR using the SYBR premix ex taq (TakaRa, Shiga, Japan). This experiment involved three separate biological replicates as well as three technical repeats. Table A4 contains a list of primers utilized in this investigation.

### 4.7. Lignin Content Determination and Histochemical Staining

A tissue staining technique was employed to analyze the potential involvement of *GhCCoAOMT7* in lignin biosynthesis in cotton stem tissues. Stem sections from cotton carrying pTRV::GhCCoAOMT7 and pTRV::00 constructs were selected to prepare slides and were mounted. The sections were then subjected to staining using a phloroglucinol lignin staining solution. During the staining process, the lignin was first acidified using a lignin acidification solution prior to staining with phloroglucinol staining solution. Subsequently, the differences in lignin deposition in the stem cortex were observed and examined under a microscope [58].

A kit was used for the determination of cellulose, hemicellulose, pectin, and lignin contents [59]. For hemicellulose and lignin determination, the stems were dried to a constant weight at 80 °C, then crushed and passed through a 50 mesh sieve, and 0.05 g was weighed and placed into 1.5 mL EP tubes. A characteristic absorption peak at 280 nm is observed after acetylation of phenolic and hydroxyl groups in lignin. The absorbance value at 280 nm is positively correlated with lignin content. Hemicellulose has a specific absorption peak at 530 nm, and its content was measured after treatment. Samples for cellulose and pectin determination were each weighed at 0.1 and 0.05 g, respectively, and after processing, their absorbance was measured at 620 nm and 530 nm, respectively, to determine the content of cellulose and pectin.

## 5. Conclusions

In summary, this study conducted a comprehensive analysis of *CCoAOMT* genes in four cotton species, resulting in the identification of 50 *CCoAOMT* genes. Based on sequence similarities, the genes were categorized into five groups by phylogenetic analysis. Analysis of exon-intron structures and conserved motifs in cotton *CCoAOMT* genes revealed their high conservation during evolution. They exhibited uneven distribution across chromosomes. Collinearity analysis demonstrated varying degrees of gene loss among the *CCoAOMT* gene family members during cotton evolution. Promoter cis-element analysis revealed the presence of regulatory elements associated with plant growth and stress responses, and transcriptomic data analysis indicated the high expression of *GhCCoAOMT7* in stems. Subcellular localization analysis of *GhCCoAOMT7* revealed its nuclear and plasma membrane localization. Silencing of *GhCCoAOMT7* resulted in decreased lignin content in cotton stems, as confirmed by lignin staining, determination of lignin content, and qRT-PCR. This study confirms the crucial role of *GhCCoAOMT7* in lignin biosynthesis in cotton and provides new molecular targets for genetic improvement and breeding strategies. Increasing lignin content can increase the stress resistance of cotton and improve fiber quality. Regulating the expression of *GhCCoAOMT7* could enable precise modulation of lignin biosynthesis and thus optimize cotton growth and yield. This finding provides an important basis for understanding the functions of *CCoAOMT* genes in plant growth and development and has significant implications for theoretical support of cotton improvement strategies.

## Data Availability

All data are incorporated into the article and its Appendix A and Appendix B.

## Appendix B

**Table A1:** The basic information and characteristics of the *CCoAOMT* genes in *G.arboreum*, *G. barbadense*, *G. hirsutum*, and *G. raimondii*.

Gene Name	Gene ID	Chromosome	AA Size	MW (Da)	Isoelectric Point	Prediction of Subcellular Localization
GhCCoAOMT1	Gh_A03G1660	A03	194	21,852.3	6.94	Cytoplasmic
GhCCoAOMT2	Gh_A04G1032	A04	248	28,009	5.97	Cytoplasmic
GhCCoAOMT3	Gh_A04G1207	A04	191	21,654.7	4.66	Cytoplasmic
GhCCoAOMT4	Gh_A04G1208	A04	234	26,668.4	4.81	Cytoplasmic, PlasmaMembrane, Chloroplast
GhCCoAOMT5	Gh_A06G0620	A06	275	31,036	7.5	Cytoplasmic
GhCCoAOMT6	Gh_A08G2183	A08	229	25,631.2	6.15	Chloroplast
GhCCoAOMT7	Gh_A12G0204	A12	318	36,179.5	9.46	Mitochondrial, Cytoplasmic, Nuclear
GhCCoAOMT8	Gh_A12G1072	A12	287	31,906.4	8.82	Chloroplast
GhCCoAOMT9	Gh_A13G0363	A13	250	28,239.2	5.18	Cytoplasmic
GhCCoAOMT10	Gh_D04G1586	D04	248	28,073	5.39	Cytoplasmic
GhCCoAOMT11	Gh_D04G1818	D04	248	28,511.8	6.69	Cytoplasmic
GhCCoAOMT12	Gh_D06G0703	D06	245	28,037.5	9.55	Mitochondrial
GhCCoAOMT13	Gh_D08G2549	D08	274	31,102.5	6.69	PlasmaMembrane, Cytoplasmic
GhCCoAOMT14	Gh_D12G1196	D12	306	34,079	9.16	Chloroplast, Nuclear
GhCCoAOMT15	Gh_D13G0408	D13	250	28,287.2	5.18	Cytoplasmic
GhCCoAOMT16	Gh_D12G0206	D12	223	25,039.7	5.36	Cytoplasmic
GaCCoAOMT1	Ga03G2453	A03	194	21,852.3	6.94	Cytoplasmic
GaCCoAOMT2	Ga04G0015	A04	241	27,513.4	4.73	Cytoplasmic
GaCCoAOMT3	Ga04G0016	A04	234	26,668.4	4.81	Cytoplasmic
GaCCoAOMT4	Ga04G0313	A04	260	29,427.8	7.13	Cytoplasmic
GaCCoAOMT5	Ga05G3423	A05	108	11,912.8	5.08	PlasmaMembrane, Chloroplast
GaCCoAOMT6	Ga06G0722	A06	269	30,296	5.85	Cytoplasmic
GaCCoAOMT7	Ga08G2876	A08	242	27,170	5.02	Cytoplasmic, Chloroplast
GaCCoAOMT8	Ga12G1724	A12	287	31,932.5	8.82	Chloroplast
GaCCoAOMT9	Ga13G0397	A13	250	28,330.3	5.91	Cytoplasmic
GrCCoAPMT1	Gorai.004G283700	D04	237	26,632.4	5.69	Cytoplasmic, Chloroplast
GrCCoAPMT2	Gorai.005G237100	D05	240	27,340.2	6.17	Cytoplasmic
GrCCoAPMT3	Gorai.008G024000	D08	318	36,161.6	9.69	Mitochondrial, Cytoplasmic, Nuclear
GrCCoAPMT4	Gorai.008G133100	D08	306	33,994.9	9.32	Chloroplast
GrCCoAPMT5	Gorai.010G081000	D10	274	30,720.3	4.97	Cytoplasmic, Chloroplast
GrCCoAPMT6	Gorai.012G149800	D12	248	28,073	5.39	Cytoplasmic
GrCCoAPMT7	Gorai.012G175500	D12	241	27,482.3	4.66	Cytoplasmic
GrCCoAPMT8	Gorai.013G045300	D13	250	28,356.3	5.39	Cytoplasmic
GbCCoAOMT1	GB_A03G2207	A03	247	28,000	6.17	Cytoplasmic
GbCCoAOMT2	GB_A04G1485	A04	248	28,025	5.97	Cytoplasmic
GbCCoAOMT3	GB_A04G1725	A04	234	26,668.4	4.81	Cytoplasmic
GbCCoAOMT4	GB_A04G1726	A04	191	21,626.6	4.42	PlasmaMembrane, Chloroplast, Cytoplasmic
GbCCoAOMT5	GB_A06G0796	A06	238	26,897.9	4.93	Cytoplasmic
GbCCoAOMT6	GB_A08G2894	A08	267	29,974	6.11	Cytoplasmic
GbCCoAOMT7	GB_A12G0214	A12	318	36,223.6	9.46	Mitochondrial, Cytoplasmic, Nuclear
GbCCoAOMT8	GB_A12G1419	A12	287	31,906.4	8.82	Chloroplast
GbCCoAOMT9	GB_A13G0414	A13	250	28,308.3	5.39	Cytoplasmic
GbCCoAOMT10	GB_D04G1874	D04	215	24,341	5.38	Cytoplasmic
GbCCoAOMT11	GB_D04G2117	D04	190	21,780.8	4.95	Cytoplasmic
GbCCoAOMT12	GB_D04G2118	D04	241	27,553.4	4.74	Cytoplasmic
GbCCoAOMT13	GB_D06G0782	D06	236	26,900.1	8.95	Mitochondrial, Cytoplasmic, Chloroplast
GbCCoAOMT14	GB_D08G2886	D08	242	27,287.2	5.39	Cytoplasmic, Chloroplast
GbCCoAOMT15	GB_D12G0230	D12	318	36,094.4	9.31	Cytoplasmic, Mitochondrial
GbCCoAOMT16	GB_D12G1416	D10	287	32,005.5	8.64	Cytoplasmic, Chloroplast
GbCCoAOMT17	GB_D13G0404	D11	250	28,356.3	5.39	Cytoplasmic

**Table A2:** The non-synonymous (Ka) to synonymous substitution ratio (Ks) in duplicated *CCoAOMT* gene pairs in cotton.

Gene Pairs		Ka_Ks	Gene Pairs		Ka_Ks
GbCCoAOMT1	GaCCoAOMT9	0.065068696	GhCCoAOMT4	GaCCoAOMT2	0.541581445
GbCCoAOMT4	GaCCoAOMT2	0.540163087	GhCCoAOMT5	GaCCoAOMT6	0.100265716
GbCCoAOMT5	GaCCoAOMT6	0.911349732	GhCCoAOMT6	GaCCoAOMT7	1.548836158
GbCCoAOMT10	GaCCoAOMT4	0.040931697	GhCCoAOMT10	GaCCoAOMT4	0.070833499
GbCCoAOMT12	GaCCoAOMT2	0.229109501	GhCCoAOMT12	GaCCoAOMT6	0.095121818
GbCCoAOMT13	GaCCoAOMT6	0.114461472	GhCCoAOMT13	GaCCoAOMT7	0.322398054
GbCCoAOMT14	GaCCoAOMT7	0.314951527	GhCCoAOMT14	GaCCoAOMT8	0.192725361
GbCCoAOMT16	GaCCoAOMT8	0.267528849	GhCCoAOMT15	GaCCoAOMT9	0.382526196
GbCCoAOMT17	GaCCoAOMT9	0.28693383	GhCCoAOMT2	GrCCoAPMT6	0.163281629
GbCCoAOMT1	GrCCoAPMT8	0.060498137	GhCCoAOMT4	GrCCoAPMT7	0.472390717
GbCCoAOMT2	GrCCoAPMT6	0.1226389	GhCCoAOMT5	GrCCoAPMT5	0.454166854
GbCCoAOMT4	GrCCoAPMT7	0.471143834	GhCCoAOMT6	GrCCoAPMT1	0.671213344
GbCCoAOMT5	GrCCoAPMT5	0.600294416	GhCCoAOMT7	GrCCoAPMT3	0.166795279
GbCCoAOMT7	GrCCoAPMT3	0.145483775	GhCCoAOMT8	GrCCoAPMT4	0.184354187
GbCCoAOMT8	GrCCoAPMT4	0.184354187	GhCCoAOMT9	GrCCoAPMT8	0.2865639
GbCCoAOMT9	GrCCoAPMT8	0.191069052	GhCCoAOMT12	GrCCoAPMT5	0.443452894
GbCCoAOMT12	GrCCoAPMT7	0.131872778	GhCCoAOMT13	GrCCoAPMT1	0.302139173
GbCCoAOMT13	GrCCoAPMT5	0.29703111	GhCCoAOMT14	GrCCoAPMT4	0.786156023
GbCCoAOMT14	GrCCoAPMT1	0.204346831	GhCCoAOMT15	GrCCoAPMT8	0.143345629
GbCCoAOMT16	GrCCoAPMT4	0.786002408			

**Table A3:** *Cis*-elements in the cotton *CCoAOMT* promoters.

Name	Sequence	Explain
GCN4_motif	TGAGTCA	cis-regulatory element involved in endosperm expression
AC-I	(T/C)C(T/C)(C/T)ACC(T/C)ACC	lignin correlation
AC-II	TCACCAACCCCC	
ABRE	ACGTG	cis-acting element involved in the abscisic acid responsiveness
AuxRR-core	GGTCCAT	cis-acting regulatory element involved in auxin responsiveness
TGA-element	AACGAC	
WUN-motif	AAATTACT	wound-responsive element
TATA-box	TATAA	core promoter element
O2-site	GATGATGTGG	
ARE	AAACCA	cis-acting regulatory element essential for the anaerobic induction
LTR	CCGAAA	cis-acting element involved in low-temperature responsiveness
CAT-box	GCCACT	cis-acting regulatory element related to meristem expression
TGACG-motif	TGACG	cis-acting regulatory element involved in the MeJA-responsiveness
TATC-box	TATCCCA	cis-acting element involved in gibberellin-responsiveness
TC-rich repeats	ATTCTCTAAC	cis-acting element involved in defense and stress responsiveness
MBS	CAACTG	MYB binding site involved in drought-inducibility
GC-motif	CCCCCG	enhancer-like element involved in anoxic specific inducibility
HD-zip	CTTTACCAACC	Palisade differentiation

**Table A4:** Primer sequences.

Primer Name	Primer Sequence	Primer Function
GhCCoAOMT2-F	CAACACCACCCAAGAGCAAC	gene clone
GhCCoAOMT2-R	TTGACACGGCGGCAAAGGG	
GhCCoAOMT7-F	GAGATGGGTCCCAGTCCAGC	
GhCCoAOMT7-R	TCATTTAACGCGACGGCAAA	
GhCCoAOMT14-F	ATGACAAAAAGCGCAGCAA	
GhCCoAOMT14-R	TCGGCATATTGTCATTCCAT	
eGFP-GhCCoAOMT2-F	GGGGCCCGGGGTCGACATGGCAACCAACACCACCC	carrier conjugation
eGFP-GhCCoAOMT2-R	TACCGGATCCACTAGTTTTGACACGGCGGCAAAG	
eGFP-GhCCoAOMT7-F	GGGGCCCGGGGTCGACATGGGTCCCAGTCCAGCT	
eGFP-GhCCoAOMT7-R	TACCGGATCCACTAGTTTTAACGCGACGGCAAAGG	
eGFP-GhCCoAOMT14-F	GGGGCCCGGGGTCGACATGGCAACCAATACGCAAGAGC	
eGFP-GhCCoAOMT14-R	TACCGGATCCACTAGTTTTGACGCGACGGCAAAG	
TRV-GhCCoAOMT7-F	TAAGGTTACCGAATTCCTCGAGACCAGTGTGTATCCGAG	
TRV-GhCCoAOMT7-R	GCTCGGTACCGGATCCAGGGCCCTCTTTGAAATCAATTTTGT	
GhCCoAOMT2-YG-F	AACACACTGTGGAATGGGTCGG	qRT-PCR
GhCCoAOMT2-YG-R	TCACCAACAGGGAGCATGCAAA	
GhCCoAOMT3-YG-F	CATTGCCTGAGGATGGCAAGGT	
GhCCoAOMT3-YG-R	AGGCATCTGAGGGGAAGAACTCA	
GhCCoAOMT7-YG-F	TGTATCCGAGGGAGCCTGAA	
GhCCoAOMT7-YG-R	GCAGACCCAGCTCGTAGTTT	
GhCCoAOMT9-YG-F	GCAACCAACAAAACAGAAGAGC	
GhCCoAOMT9-YG-R	TTGAGGCGACGGCAAAGG	
GhCCoAOMT10-YG-F	AGAGCTCAGAGAGTTGACCGCT	
GhCCoAOMT10-YG-R	TTGTGTGCAACGCCAGCTTTTT	
GhCCoAOMT14-YG-F	GCAACCAATACGCAAGAGCA	
GhCCoAOMT14-YG-R	GACGCGACGGCAAAGGGTGA	
